# EMERGENCY DEPARTMENT USE FOR DENTAL PROBLEMS AMONG MEDICARE FEE-FOR-SERVICE OLDER ADULTS IN THE U.S. (2016 TO 2020)

**DOI:** 10.1093/geroni/igac059.2877

**Published:** 2022-12-20

**Authors:** Roshani Dahal, Stephanie Jarosek, Beth Virnig

**Affiliations:** University of Minnesota, Twin Cities, Minneapolis, Minnesota, United States; University of Minnesota, Minneapolis, Minnesota, United States; University of Florida, Gainesville, Florida, United States

## Abstract

Medicare Fee-for-Service (FFS) does not include dental coverage for older adults (65 years of age and older) but does cover emergency visits for dental problems. This study leverages Medicare Limited Data Sets to examine the use of emergency department (ED) for preventable, non-traumatic dental conditions (NTDCs) among Medicare FFS older adults from 2016 to 2020. Nationally, ~43.6 million beneficiaries sought care at the ED (average: ~8.7 million annually). Among these, ~550,000 ED visits (1.27%; ~110K annually) were for NTDCs as one of the diagnosis codes and ~200,000 ED visits (0.45%; ~40K annually) were for NTDCs as a primary diagnosis. Approximately, 5–6% of older adults with ED-NTDCs have multiple visits (94% with 1 ED-NTDC visit annually). Rates were similar in most years; however, ED use was lower in 2020 (COVID-19 pandemic). The most common diagnosis reasons include periapical abscess (tooth infection), sialadenitis, dental caries, jaw pain, and lesions of oral mucosa. Younger (65 to 74 years) and Black older adults were more likely to have primary ED-NTDC visits. Medicare paid ~$190 million for ED-NTDC visits (average: $38 million annually). Costs vary by inpatient (9%) and outpatient visits (91%). For ED-NTDCs as a primary diagnosis, the average Medicare payments for outpatient and inpatient visits were approximately $330 and $8,100, respectively. ED use for NTDCs indicates inappropriate use of valuable resources, because care provided in the EDs is incomplete (e.g., antibiotics, pain medication). Lack of follow-up with a dentist likely results in return ED visits, thus increasing costs to beneficiaries and public programs.

